# The CHAP-EMS health promotion program: a qualitative study on participants’ views of the role of paramedics

**DOI:** 10.1186/s12913-016-1687-9

**Published:** 2016-08-24

**Authors:** Madison Brydges, Margaret Denton, Gina Agarwal

**Affiliations:** 1 Department of Health, Aging & Society, McMaster University, 1280 Main St W, Hamilton, ON Canada; 2 Department of Family Medicine, David Braley Health Sciences Centre, McMaster University, 100 Main Street West, 5th Floor, Hamilton, ON L8P 1H6 Canada; 3 Department of Clinical Epidemiology and Biostatistics, McMaster University, 1280 Main St W, Hamilton, ON Canada

**Keywords:** Community paramedicine, Qualitative research, Paramedic roles

## Abstract

**Background:**

Expanded roles for paramedics, commonly termed community paramedicine, are becoming increasingly common. Paramedics working in community paramedicine roles represent a distinct departure away from the traditional emergency paradigm of paramedic services. Despite this, little research has addressed how community paramedics are perceived by their clients.

**Methods:**

This study took an interpretivist qualitative approach to examine participants’ perceptions of paramedics providing a community paramedicine program, named the Community Health Assessment Program through Emergency Medical Services (CHAP-EMS). Both participant observation and semi-structured interviews conducted with program participants were used to gain insight into the on-the-ground experiences of the program. Thematic analysis was employed to analyze all data.

**Results:**

Three themes emerged: i) Caring and trusting relationships; ii) paramedics as health advocates; iii) the added value of EMS skills. Paramedics were perceived by residents as having dual identities: first in a novel role as health advocates and secondly in a traditional role as emergency experts despite lacking contextual features associated with emergency response.

**Conclusions:**

From this exploratory, qualitative study we present an emerging framework in which to conceptualize paramedic roles in community paramedicine settings. Future research should address the saliency of these roles in different contexts and how these roles relate to paramedic practice.

**Electronic supplementary material:**

The online version of this article (doi:10.1186/s12913-016-1687-9) contains supplementary material, which is available to authorized users.

## Background

Both within Ontario and internationally, the role of Emergency Medical Services (EMS) is expanding to address a range of health problems in both community and institutional settings. The management of low acuity illnesses in the community has been broadly defined as ‘community paramedicine’ [1]. While this definition remains ambiguous in nature, community paramedicine programs often feature paramedics working in alternative care models or with broadened scope of practices. Although the literature on community paramedicine remains limited, there is evidence to suggest that paramedics are uniquely positioned to provide health care in the community and may be important actors in addressing broader, systemic health care system problems, such as hospital overcrowding [1, 2]. This shift in paramedic roles may also be reflective of health care policy reforms in Ontario, including aims to improve primary care through the development of integrated health care teams and a focus on community based care.

Not only in the Ontario context, but also throughout Canada, the development of community paramedic roles is relatively new and represents a distinct departure away from the dominant emergency medical paradigm of EMS. Traditionally, paramedics are trained to respond to emergencies in the community that require acute interventions. However there has been an increased recognition that paramedics respond to a large number of low acuity patients, and these patients could be better served outside of the acute care system [3]. A clearer definitions of paramedics’ roles are needed to assist the planning and staffing of different types of community paramedic program across Canada, and the interdisciplinary interactions that occur due to these.

Defining the role of paramedics in alternative settings it is also necessary to develop roles that complement other health care providers and align with relevant policy goals. From the patient perspective, understanding how paramedics are perceived as health care providers will assist in clarifying what their role should be in novel health care settings. Consequently, we identified the need for research in the Ontario context to examine these gaps. This research took an exploratory, qualitative approach to examine older adults’ experiences of a community paramedicine program called the Community Health Assessment Program through Emergency Medical Services (CHAP-EMS). This study sought to understand participants’ perceptions of paramedic providers to explore their role in this unique practice setting and ultimately create an emerging framework to examine paramedic roles in community paramedicine programs.

## Methods

### Study setting

The CHAP-EMS program [4] is a multifaceted cardiovascular and diabetes community health prevention program operating since 2013 in a large municipality in the Greater Toronto Area. The program was delivered by modified paramedics to residents living in a subsidized housing building identified as having a large 911 call volume. Approximately 260 seniors resided at the building at the time of the study.

Modified paramedics refers to paramedics who cannot clinically practice ‘on the road’ (on an ambulance), for example due to an injury. The paramedics were not responsible for responding to emergency 911 calls during the CHAP-EMS sessions.

The CHAP-EMS program was delivered on a weekly basis to building residents, who could have their cardiovascular health risks assessed by the paramedics once a week during an intervention session. The sessions were held in a common room in the building and consisted of a semi-private area where paramedics would assess participants and a casual lounge style “waiting room” where participants could wait to be seen by the paramedics. This provided the ideal opportunity to observe both individual level interactions with paramedics as well as group dynamics between paramedics and participants in the waiting area. This study occurred as part of a larger investigation into the effects of the CHAP-EMS program [5]. Based on data from the study period, March to June, 2014, there were 1,365 participant visits to the intervention session by 79 participants. Participants were 68 % female, 90 % had family doctors and 87 % were over the age of 65.

### Study design

We used an interpretivist qualitative study design that included participant observation and semi-structured interviews. An interpretivist approach seeks to understand the actions and perspective of individuals through their own worldview and within their social context [6]. Given the ethnographic approach and goal of this study, this lens was ideal in examining how participants experiences and perceptions shaped their view paramedics. Ethics approval was obtained from the Hamilton Integrated Research Ethics Board.

### Researchers’ background

The field-researcher (MB) was a health studies graduate student, with experience conducting qualitative research. She is also a practicing paramedic in Ontario, however not in the geographical region in which the research took place. MB conducted both the participant observation and interviews for the purposes of completing an M.A. thesis. GA is a practicing family physician with a research background in epidemiology, and is the principal investigator of the CHAP-EMS study. MD is a sociologist in the Department of Health, Aging and Society and she and GA co-supervised the research project.

### Sampling

This study used purposive sampling which included adults living in the residential building in which CHAP-EMS took place including those who were currently participating in the CHAP-EMS program. To ensure we gathered data from a wide range of participants, we included participants with variances in age, gender, years in the program, attendance at the program, and health status. The number of interviews and length of the observational period was not pre-determined but occurred until thematic saturation was met. In total, of the 79 participants of the program, semi-structured interviews were conducted with 15 participants.

### Observational period

The observational period focused on the residents who attended the CHAP-EMS program and the paramedics operating the sessions. Due to the setting of the CHAP-EMS program, both the waiting room area and the semi-private section where the health checks took place provided an opportunity to observe the social dynamics of the programs. Preliminary field work highlighted that the participants of the program and the paramedics had prolonged social interactions before and/or after the sessions. During this time it was observed that some participants attended the sessions in order to socialize and not have their health check done by the paramedics. Thus it was the purpose of including observational methods in order to gain detailed descriptions of the on-the-ground, daily lived experiences of the participants attending the program that went beyond solely the health care interaction between the participants and paramedics. The type of thick description and level of depth provided from physically *being there* would not have been possible using strictly qualitative interviews [6].

In total, ten CHAP-EMS sessions were attended from March to June, 2014, totaling 80 h of field work. The participant observation conducted in this study was by a single researcher (MB). Residents were made aware of the study’s purpose and the researchers’ role. The researcher took an active role in the observational sessions, accompanying the paramedics as they performed tasks required for the program. Questions were asked to both paramedics and participants except when participants were receiving their individual health check due to patient privacy. No notes on patient care or health problems were taken.

Short observational notes were kept during the sessions with detailed field notes written following the individual sessions. The observational notes were guided by the following topics: length and type of interactions amongst paramedics and participants; interactions between participants; interactions between paramedics and allied health care workers or community services; behaviors and actions of participants while waiting to attend session. Field notes remained broad to include a detailed descriptions of the daily interactions and activities that occurred at the CHAP-EMS sessions, including the researcher’s own reflections from the sessions.

### Semi-structured interviews

To complement the observational data, semi-structured interviews were conducted with residents who participated in the CHAP-EMS program. Participants were asked to take part in an interview following attending a CHAP-EMS session. During the study period, fifteen semi-structured interviews were conducted with building residents. The interviews were conducted over the course of the observational period to pursue emerging themes as they arose. As the interviewing researcher (MB) attended the sessions on a weekly she became familiar with building members and was able identify residents who attended the program on a regular or sporadic basis. Following attending the session, residents who both attended the sessions weekly as well as on a sporadic basis (once to twice a month) were asked to take part in an interview. All residents asked to partake in an interview agreed. Interviews took place in a private room or in the participant’s home following the session. Following obtaining consent, all interviews were audio-recorded and transcribed for analysis. Demographic information was collected for descriptive purposes and to ensure a wide range of individuals were interviewed.

Using open ended non-judgmental questions and probes, participants were asked to describe and reflect on their experiences in the CHAP-EMS program. An interview protocol and interview guides were used to assist the process (see Additional file 1). Questions were piloted in two interviews to assess clarity, intent, potential biases, and appropriateness. This process allowed the interviewer to practice (e.g., avoiding persuasiveness), adapt, and refine the interview process as necessary [7]. As new themes emerged from the participant observation and interviews, new questions were generated and pursued in subsequent interviews.

### Analysis

The researchers employed thematic analysis to analyze the interview transcripts and observational data, because this methodology takes an inductive, data driven approach to identify, analyze, and report patterns within the data [8]. This approach was ideal given the goal of this research to inform a framework to understand paramedic roles in novel settings. This approach allowed for flexibility in expanding on new themes as they emerged.

The interviews were first transcribed verbatim, and any initial thoughts or ideas were recorded. Next, the data was coded independently by MB and GA. Following this step, the codes were generated into potential themes and a thematic map of all the themes was created. Following this, the themes were then defined, organized categorically, reviewed repeatedly, and re-conceptualized until the final themes were produced. Any inconsistencies between the coders were solved by joint discussions amongst all authors.

In this study, transcripts were reviewed with the audio files to ensure they did not contain errors that occurred during transcription. Frequent meetings were held to discuss the coding process and clarify any disagreements on the codes. Both researchers coded the transcripts independently and came to the final themes based on joint agreement, further contributing to the trustworthiness of the results [7].

## Results

Nine females and six males were interviewed, with ages ranging from 63–89. Only three participants lived with a spouse. Twelve participants had hypertension, and there were multiple participants with chronic health conditions (such as COPD, diabetes). The remainder of participants had minor health problems. All participants had at least one medical problem. Thirteen participants had been attending the program for more then 2 years.

From this study, three key themes emerged which will be discussed in depth next: i) trust, care and respect; ii) paramedics as health advocates; iii) the added value of EMS skills.

***1. “We know they care”: Trust, care and respect***

The participants of the CHAP-EMS program had trusting and close relationships with the paramedics operating the program. Participants expressed that in contrast to the impersonal experiences they had with many other health care providers, the CHAP-EMS sessions were an individualized experience. Participants attributed this to the personal characteristics of the paramedics, such as their welcoming, caring, and thoughtful attitude. Participant 107 stated:

“It has a lot to do with personality this thing. And how he interacts with people. Because he is open, is caring, is fun, he likes the people. He is concerned. All the things you want in a doctor or a health care professional. It’s not the pills they give you; it’s the feeling they give you.”

The relationship between participants and paramedics was in part due to the care, support and guidance they received regarding their health, but also from their interactions with the paramedics unrelated to their health. Many participants stated that while attending the sessions they could socialize and have fun with the paramedics alongside discussing their medical concerns. The residents would frequently hug the paramedics, thank them for their time, and refer to them as “good friends”. Residents frequently brought down personal items, such as favorite books, photo albums of family members, or things they thought the paramedics would enjoy, to share with them. One participant stated:

“I think everyone is doing an excellent job. That is what they are here for right? You know, and most of them are more then that. They are like a family right? And that’s how they make everyone feel.”-Participant 6

For some participants, their relationship with the paramedics allowed them to discuss problems they had not brought up to other health care providers, such as their mental health. Participant 12 explained:

“I was ashamed and I would not talk about it. Then [the paramedic] and I were talking and it sort of came out. I feel comfortable knowing that someone knows what is happening. Because in case I don’t come down [to the program] then they will know what is happening.”

Many of the participants of the CHAP sessions were weekly visitors of the program, regardless of their health status. For some residents, they did not only attend the CHAP sessions to have their blood pressure checked or for follow up on a health concern (in some cases it was not a part of the visit at all), but rather to socialize with the paramedics or follow up on an event or initiative occurring in the building. Participants spoke robustly of the welcoming, open attitudes expressed by the paramedics operating the program. In turn and over time, these relationships allowed participants to have a positive health care experience and develop trusting, caring relationships with the paramedics. These relationships extended beyond individual’s health to broader events and initiatives occurring in the building.

***2. “It’s nice knowing someone is there”: Paramedics as health advocates***

While it was the goal of the CHAP-EMS program to address individuals’ health and wellbeing through a health awareness program, the program expanded naturally to address much more then this primary goal. A discourse emerged in which paramedics were viewed as advocates for both the entire building and resident’s individual health. Paramedics were viewed as important actors who supported elderly or frail residents in the building, and enhanced feelings of safety and security on a broader level.

Residents expressed that the paramedics provided a sense of support and security for residents in the building that they did not have before. Paramedics operating the program invested a significant amount of time to connect with other service providers in the building, and this in turn resulted in many positive outcomes for residents. Participants stated that they felt a safety net was now in place in the building and it was reassuring knowing someone was taking care of them. One participant stated:

“I am glad they are here. It seems like people are at least looking after us. More then what the other people seem to want to do.”–Participant 103

Participants also felt that the presence of the paramedics weekly in the building enhanced feelings of support for aging residents. One participant stated:

“I think once people become seniors, or even 60 for that matter, having a system like this in any seniors building, is more valuable then anything because of checking blood pressures, stuff like that, the continuation. It’s not your standard stuff that gets done every week. It’s the other things that are going to be the biggest things. And without that people are going to get worse, their conditions are going to get worse. But it really extends the well being of the seniors, in every way. Medically, emotionally, because there is an emotion thing to say someone is there and you can go talk to them.”–Participant 3

On an individual level, residents valued the paramedics’ presence in the building as many had poor health or were concerned about requiring more help in the future. The paramedics were a source of support both physically and mentally for residents. Participant 2 described when asked if he would take his blood pressure at home instead of at the program:

“No. I wouldn’t want to do it. Not everyday. Just in case its off, then I go, oh what am I going to do? Because there is nobody here to support me now. When you have these guys here, you feel different because anytime you have a problem you know those guys are just behind that door.”–Participant 2

Paramedics also assisted in advocating for participants’ health, in turn making them more aware and involved in managing their health. Paramedics provided a combination of knowledge and advice for residents, such as explaining medications, health procedures or seeking health care from other providers such as specialists. One—participant stated:

“It keeps you involved in your own health management also right. It brings awareness of health issues. I know I have gone through it already where I came down here and my pressure had dropped really low and right away the paramedic made an appointment for me. He called the doctor and made an immediate appointment for me. You know? So its things like that that you become aware of. That helps a lot of people in that way.”—Participant 6

For residents that had complex medical histories or complex social problems, the paramedics often took a significant amount of time to collaborate with family physicians, and other health care providers, both within the building (such as a diabetic clinic) and in the community, directly interacting with Community Care Access Center’s, case managers and other local social services. This could in some instances be a timely process and span over weeks before the issue was resolved. The paramedics would often “report” these updates to the other paramedics operating the program in order for the issue to continue being addressed in subsequent CHAP sessions.

Additionally paramedics become involved in organizing other initiatives in the building, such as helping some residents sell healthy food to other building residents and assist with organizing social activities such as a gardening club. Thus paramedics’ role as health advocates encompasses a holistic view of health, in which other activities such as assisting with dietary concerns and providing opportunities for social engagement were included.

Paramedics as health advocates emerged as a natural progression of the goals of the CHAP-EMS program. However, an unexpected theme arose from the interviews in that paramedics were valued for their emergency skill set, which will be discussed next.

***3. “They saved my life”: the added value of EMS skills***

A salient theme from the interviews was that residents valued paramedic skills that were not the focus of the CHAP-EMS program. The paramedics were situated as part of a health prevention/promotion role and separated from their emergency role, such as an ambulance and standard equipment. However, elements of paramedics’ “emergency” role remained important and valuable to residents. That is to say that while the paramedics could not carry out their normal “emergency” procedures, their ability to identify and act in the face of an acute medical event was a highly valued skill by the residents. A common discourse emerged in which participants spoke of the paramedics “saving” themselves or other residents in the building:

“I guess the biggest, biggest thing that I have heard time and time again that they have stepped in and directly or indirectly saved people. They have helped a lot of people, more then one, where it was life or death. Who knows where they would be now. Forgive me, mine was just a choking. Theirs [another resident] was major surgery. Who knows where they would be?”—Participant 3

“Well they helped some people who had to go to the hospital right away. And it feels really good because I might need it one day. So they have saved some lives in here. And that is worth anything. That is worth millions of dollars. So that is what I find really good.”—Participant 4

In addition, residents valued and respected that the paramedics would at times encourage them to seek medical attention, even when they were reluctant to. Participants spoke of paramedics advocating them to be more proactive about their health, and in some cases this led to life-saving procedures being carried out. One participant explained:

“They saved my life really. About end of August, all of the sudden I couldn’t walk. And the paramedic said, come on, lets go, I have to drive you to the hospital. And they drove me to the hospital and I tell you I was thankful for that. They pushed me to go. And then I had two stents put in. They saved my life.”—Participant 1

Residents took comfort in knowing that the paramedics would step in in the case of an emergency. Participants also expressed that they valued the emergency medical knowledge and experience the paramedics had, and their timely assessment of medical conditions:

“So, they in some cases, because of hands on experience, on certain issues they may even know more then doctors do. Right then and there, doctor doesn’t see them, they see them when they are surviving, we’ll go give them an operation or something. These guys see a lot.”—Participant 3

“And they can pick up really quickly. They have done it a few times when something has been wrong. And that’s invaluable. I think that’s three or four people they have taken out on ambulance on their recommendations.”—Participant 9

Although paramedics were not in their traditional role operating the CHAP program, they retained the many features traditional to EMS, such as the ability to “save lives” and act quickly during an acute medical situation. This was highly valued by residents, who openly discussed their own experiences or of others in the building who required immediate intervention on behalf of the paramedics. Thus a large proportion of the emergency medical paramedics skill set, although were not the focus of the program, had immeasurable value to the building residents.

## Discussion

This study analyzed older adults’ perceptions of paramedics’ roles in a community health promotion program. A number of key findings emerged: paramedics were viewed as caring, respectful and trustful health care providers; and paramedics had a dual role as both health advocates and as emergency experts. Our findings speak to a gap in the literature on how paramedics are perceived by the users of community paramedicine programs. As patient centered approaches become more prominent [9] and as paramedic roles change across North America, research needs to address how paramedics are perceived as health care providers, particularly in novel settings.

A recent study examining a community paramedicine program operating in rural Ontario supports the findings of this research in that paramedics improved health care access, increased feelings of security and support for vulnerable residents (although poorly defined in the study), and empowered patients to better manage their own health [10]. Paramedics were viewed as friendly and trustworthy health care providers that filled a much needed gap in health care services to a rural area. Our results are consistent with these findings given that the community paramedicine program researched here occurred in an urban area. The task of empowering patients also requires paramedics to be viewed positively, with a trusting relationship, again confirming our study’s results.

From this work we can create a theoretical framework to describe paramedic roles from the patient/client perspective, that is useful in the broader context of community parmedicine in Canada and beyond. Three areas suggest an emerging framework to understand paramedic roles: firstly, in relation to existing constructs of patient-health care provider interactions; secondly, to the concept of advocacy, which has been studied in depth in the fields of nursing and medicine; and lastly, possibly unique to paramedics, the concept of a dominant ‘emergency expert’ identity.

### An emerging theoretical framework of paramedic roles

The first component of understanding paramedic roles is their relationship with their patients. Patient and health care provider interactions have long been studied in the fields of medicine, particularly involving nurses and physicians. Early research conceptualized these relationships focusing on the power dynamic between health care providers, who often hold professional power and control over their patients [11]. Theories of patient-health care provider interactions involve the negotiation of power, the assumption of specific roles, communication, and ethics. Several models have been proposed to explain health care provider and patient relationships. Based on a synthesis of qualitative research examining physician-patient relationships, Ridd and colleagues [12] propose a model in which relationships are built on positive experiences with a physician, developed over time (seeing the same physician again) and impacted by feelings of trust, regard, and knowledge. All elements of this model encompass aspects of effective communication, such as listening well and demonstrating a caring attitude. Similarly, responsive relationships in nursing (defined as relationships that result in improved patient outcomes) have been theorized as ones that encompass trust, mutuality, and respect [11, 13].

In this study we found that paramedics had dual roles as both health advocates and emergency experts. The caring, trustful and respectful relationships paramedics built with patients impacted the development of healthy patient-paramedic interactions. In the case of CHAP-EMS, paramedics took on a new advocacy role that allowed them to spend more time with individuals, advising about lifestyle and health promotion, and become somewhat integrated into the building community. This role had a direct effect on their relationships with patients. First, several paramedics staffed the CHAP-EMS program for an extended period of time. This allowed for both continuity in care and the formation of trusting relationships between participants and paramedics. Apparent in this process was the importance of how the paramedics interacted with participants. The paramedics had warm, caring attitudes, listened well, and advocated for participants to become more involved in their health. Paramedics also participated in a number of social activities with building residents, and it was expressed by participants that they felt the paramedics “knew them” on an individual level.

One element of the paramedic-patient relationship that needs to continue to be explored further is the issue of continuity. The paramedics operating in this iteration of CHAP-EMS were not permanent, and were required to return to active duty on the ‘road’ once their period with CHAP-EMS was over. During the study period, several paramedics spent an extended period of time operating the program, including one paramedic who had been with the program from its inception. Whether positive relationships between paramedics and participants were a consequence of the amount of time spent by these individuals together or not needs to be explored further, and could have an impact on future community paramedicine programs in different settings. It is highly likely that participants could become attached to a particular paramedic, recognizing their physical and social attributes and therefore feeling comfortable with them over time. This is known to happen with other health care professionals as well, including physicians [12].

Paramedics also took on a strong advocacy role. In the CHAP-EMS context, paramedics were able to advise and empower patients, and when necessary advocate for them in their negotiation of the complex health care system. Paramedics’ roles were shifted from being strictly emergency experts, to that of ‘health advisors’ and ‘advocates’. The premise, structure, and setting of the CHAP-EMS program reinforced that the program was there to help residents be more proactive regarding their health and provided a safe environment to engage at times, in sensitive topics relating to their health and wellbeing. This is in keeping with literature that states paramedics engage in advocacy due to a sense of professional obligation [14].

These professional obligations and roles are not unique to EMS, but rather are the moral and ethical responsibilities associated with all health care professions to do what is best for their patients [15]. Advocacy has always been seen as a core role for family physicians, and is one of the pillars of Canadian Family Medicine [16]. Advocacy is also seen as a core role for other health care providers such as nursing [17] and social workers [18]. Advocacy reflects the ethical and moral obligations of health care providers in representing the needs or requests of patients in order to preserve their autonomy [13, 16]. While no universal definition exists, advocacy in health care has been defined as providing support for patients in decision making, and representing the patient’s concerns or wishes to other health care providers/groups [18]. The concept of paramedics as advocates on a broader level has emerged in this study. That is, these paramedics went beyond addressing individual health needs to become active in inducing broader changes within the building, though maintaining their own professional boundaries and professional identities in doing so.

Lastly, paramedics retained a strong ‘emergency’ identity that was highly valued by participants. Given the contextual setting of CHAP-EMS and clear program goals unrelated to emergency medicine, it is interesting that paramedics were still defined in this way. Paramedics were actively involved during an emergency in the building, despite lacking the associated equipment and an ambulance. Through this, paramedics’ emergency identities were socially reinforced by the “story telling” amongst building residents when paramedics intervened in an emergency situation. Further, all of the emergency situations resulted in favorable outcomes for the individuals involved, and may have strengthened these attributes and global feelings of trust regarding paramedics as a profession, not just the individual paramedics in this study.

We present here an emerging framework in which to consider paramedic roles in community paramedicine settings in the future. Many attributes of this framework are found amongst other health care providers (i.e. trusting relationships and patient advocacy). On one hand this supports the development of community paramedicine programs as paramedics share attributes consistent with other health care providers in these roles; on the other it could also mean other health care providers could be substitutes for paramedics in these settings, though this would require more investigation.

In this community paramedicine program, CHAP-EMS, paramedics retained a robust emergency identity. This finding is consistent with additional literature in which the strong emergency identity of paramedics is seen as a possible barrier to the expansion of community paramedicine roles as communities and paramedic culture itself is dominated by an emergency medicine framework [19]. As seen here, paramedic’s skills to handle an emergency were both utilized (unintentionally) and highly valued by building residents. Future research should address whether this defining feature hinders the development of community paramedicine roles, or translates into “added value” for communities and community based programming such as CHAP-EMS.

### Limitations

CHAP-EMS occurred in only one residential building at the time of this study, and research is still underway to determine its function and operation in other buildings with different demographics and different paramedics. However the goal of this study was to present an emerging framework for paramedics roles in community paramedicine studies which should be explored in future research. A large limitation of this study is that no interviews were conducted with residents who spoke no English, due to the lack of a translator. Although attempts were made to interact with this group of non-English speakers, they were unsuccessful and it is unknown how this could have affected perceptions and experiences of the program.

Given the nature of the study design it is possible that not all elements of the phenomenon were recorded during the observation process. To adjust for this limitation, an extended period of time was spent in the field taking observational notes to allow time to develop an in-depth understanding of the dynamics of the program, follow up on emerging themes, and give credibility to the findings [7]. Additionally, regular meetings were held with all members of the research team to review the observational notes and follow up on emerging themes as required. Data was discussed with the research team members (GA and MD) and the consistency of interpretation was ensured by this approach.

## Conclusion

This exploratory, qualitative study of a community paramedicine program highlights from the patient perspective how a community health program, CHAP-EMS, operates on the ground and provides insight into how paramedics interact with building residents and are perceived in this setting. This study found that paramedics had dual roles as advocates for health and wellbeing and as experts in providing emergency care. Results from this study have informed an emerging framework for understanding paramedic roles in community paramedicine settings in multiple contexts: paramedics as trusting health care professionals; paramedics as patient advocates; and lastly, paramedics as emergency experts. Future research should address whether these results are generalizable to other community paramedicine programs in different settings and with different populations who may hold different views.

## Additional file

Additional file 1: Interview Guide for Semi-Structured Interviews. This document provides a list of the questions that guided the semi-structured interview process. (DOCX 12 kb)
