# Supplementary material for: The CHAP-EMS health promotion program: a qualitative study on participants’ views of the role of paramedics
Source: BMC Health Serv Res. 2016 Aug 24;16(1):435. doi: 10.1186/s12913-016-1687-9 (PMC4997682; doi:10.1186/s12913-016-1687-9)
Supplement: Additional file 1: — Interview Guide for Semi-Structured Interviews. This document provides a list of the questions that guided the semi-structured interview process. (DOCX 12 kb) [file 12913_2016_1687_MOESM1_ESM.docx]

Supplementary File 1: Interview Guide for Semi-Structured Interviews

1. How has the program impacted your use of your family doctor and other health care providers?
2. How has the program helped you access other community services?
3. What do you find most valuable about the program?
4. What was the least valuable aspect of the program?
5. Why do you participate in the program?
6. What barriers hindered you from going to the program?
7. How often do you feel you need to go?
8. How has the program changed your usage of 911 services?
9. How has the program changed your feelings of safety and security in your building?
10. Has the program changed your life style behavior (which ones)?
    1. If so, in what ways?
    2. If not, why not?
